# Methylene-Blue-Encapsulated Liposomes as Photodynamic Therapy Nano Agents for Breast Cancer Cells

**DOI:** 10.3390/nano9010014

**Published:** 2018-12-23

**Authors:** Po-Ting Wu, Chih-Ling Lin, Che-Wei Lin, Ning-Chu Chang, Wei-Bor Tsai, Jiashing Yu

**Affiliations:** Department of Chemical Engineering, National Taiwan University, Taipei 103, Taiwan; r05524112@ntu.edu.tw (P.-T.W.); r06524045@ntu.edu.tw (C.-L.L.); d01642002@ntu.edu.tw (C.-W.L.); r05524045@ntu.edu.tw (N.-C.C.)

**Keywords:** liposome, methylene blue, breast cancer cell, photodynamic therapy, zwitterion

## Abstract

Methylene blue (MB) is a widely used dye and photodynamic therapy (PDT) agent that can produce reactive oxygen species (ROS) after light exposure, triggering apoptosis. However, it is hard for the dye to penetrate through the cell membrane, leading to poor cellular uptake; thus, drug carriers, which could enhance the cellular uptake, are a suitable solution. In addition, the defective vessels resulting from fast vessel outgrowth leads to an enhanced permeability and retention (EPR) effect, which gives nanoscale drug carriers a promising potential. In this study, we applied poly(12-(methacryloyloxy)dodecyl phosphorylcholine), a zwitterionic polymer-lipid, to self-assemble into liposomes and encapsulate MB (MB-liposome). Its properties of high stability and fast intracellular uptake were confirmed, and the higher in vitro ROS generation ability of MB-liposomes than that of free MB was also verified. For in vivo tests, we examined the toxicity in mice via tail vein injection. With the features found, MB-liposome has the potential of being an effective PDT nano agent for cancer therapy.

## 1. Introduction

Recently, breast cancer is a severe disease, especially among women [1]. Similar to most types of cancer, surgery is the most commonly used treatment. The long recovery period and invasiveness of surgery are the challenges for both the doctors and the patients; hence, we wanted to develop a non-invasive agent for breast cancer treatment. The mammary cancer cell, 4T1, derived from mice, is a widely used breast cancer cell for both in vitro cultivation and in vivo tumor model [2,3]. In addition, it is used in various research aspects of breast cancer including its regulation, metastasis, and therapy [3,4,5].

Photodynamic therapy (PDT) is one of the promising cancer treatments because of its noninvasiveness and high efficiency, and it has already been proved effective in various types of cancers [6,7,8,9,10,11]. PDT triggers cell death by the reactive oxygen species (ROS) generation after photosensitizers receive light. Photosensitizers can obtain energy by absorbing light at a wavelength corresponding to the absorbance optima and initiate further reaction, including ROS production, which has been confirmed to be involved in several vital regulatory pathways [12,13]. However, there are several problems in traditional PDT resulting in low efficacy including limited penetration depth of light, low concentration in the affected area, and poor permeability of photosensitizers [14,15,16].

To lengthen the penetration depth, the absorption wavelength of photosensitizer is a critical issue to be addressed. There are two regions of “near-infrared (NIR) windows” that are usually considered because of the low endogenous absorption of biological environment, including water and pigments [17]. Methylene blue (MB), a phenothiazinium-based photosensitizer, is widely used in various applications, including anti-bacterial, toenail onychomycosis, and several cancer treatments [3,18,19,20,21]. It has an absorbance at 650–670 nm, which is coherent with the NIR window I (600–900 nm), that can be effectively activated in a deeper area [22,23]. Additionally, some research has identified that the combination of MB and photodynamic therapy is a moderate therapeutic method that can trigger cell death via apoptosis, a programmed and less stressful death, instead of necrosis [24].

Nanoparticles have been widely researched in biomedical applications in recent years because of its special characteristics [25,26,27,28]. It has been proved that particles in nanoscale can not only prolong the circulation time in body but also enter the cells via endocytosis [29]. In addition, nanoparticles are suitable for cancer therapy. Since the nutrient demand of tumor tissue is much more than normal tissue, one feature of tumor tissue is fast vascularization, which leads to poor alignment of fibroblasts and endothelial cells. The vessels consisted of poorly aligned cells that are defective and have leakage through which nanoparticles can easily enter and accumulate, and this phenomenon is known as enhanced permeability and retention effect [30].

**Figure 1:**
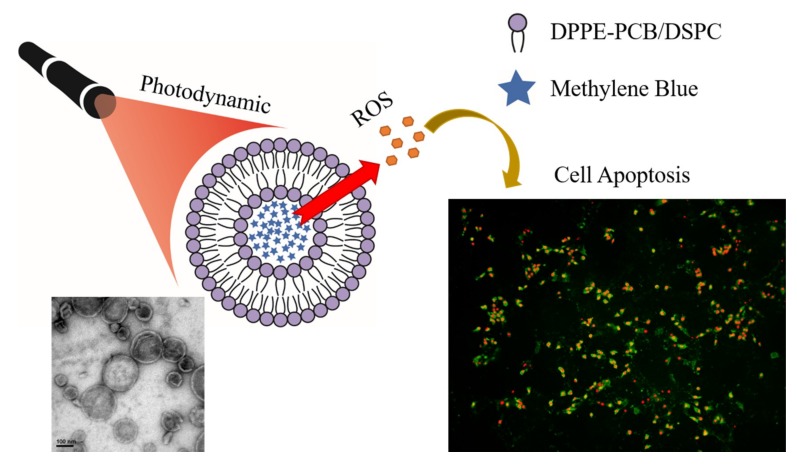
Scheme of the experiment.

Researches of nanoparticles in biomedical application can be divided into several categories such as quantum dots, magnetic nanoparticles, polymer nanoparticles, and metal polymers [19,31,32,33,34]. Among all the nanomaterials, organic nanoparticles are a branch that is popularly applied in photodynamic therapy [35]. In this study, we applied a liposome that consisted of zwitterionic polymer-lipid as a drug carrier, for which the cellular uptake mechanism was already studied [36]. Liposomes were first introduced in the mid-nineteenth century, which were constructed of the lipid bilayer mimicking the cell membrane by using similar materials [37]. With a high biocompatibility and the ability to encapsulate either hydrophobic or hydrophilic pharmaceutic ingredients, liposomes are flexible and tunable in order to fit various uses [18,38,39,40]. In order to prolong the circulation half-life of liposomes, Polyethylene glycol treated (PEGylated) liposomes were developed, and they showed an enhanced blood circulation via bypassing digestive tract [41,42]. However, PEGylation also obstructs the interaction between liposomes and tumor, lowering the uptake of liposomes. Thus, zwitterionic liposomes that can protect cargoes, prolong circulation, and improve cellular uptake were utilized [43]. In this work, we successfully encapsulated MB into the liposomes by a simple hydration and extrusion to unify the size of liposomes. The MB-liposomes were uniform in size and highly stabile. The in vitro ROS generation ability was also enhanced due to the fast uptake and concentrated photosensitizer in cells, leading to a higher cytotoxicity after irradiation. Despite the higher toxicity, the cell death pathway remained apoptosis. The MB-liposomes were also proved to be harmless by basic in vivo toxicity assessment (Figure 1).

## 2. Materials and Methods

### 2.1. Materials

Poly(carboxybetaine) modified 1,2-dipalmitoyl-sn-glycero-3-phosphoethanolamine (DPPE-PCB) were synthesized via NHS-PCB-tBu conjugated with DPPE in accordance with our previous method [39] (structure was shown in Figure S1), 1,2-distearoyl-sn-glycero-3-phosphocholine (DSPC) (Avanti 850365, Alabaster, AL, USA), Methylene blue (MB) (Alfa-Aesar A18174, Lancashire, British), sodium dodecyl sulfate (SDS) (Acros Organics 230420250, Geel, Belgium), *N*,*N*-dimethyl-4-nitrosoaniline (RNO) (Sigma D172405, St. Louis, MO, USA), imidazole (Sigma I0250), Dulbecco’s modified Eagle’s medium−high glucose (DMEM-HG) (Thermo Hyclone SH30003.02, Waltham, MA, USA), fetal bovine serum (FBS) (Biological 04-001-1A), antibiotic-antimycotic solution (Penicillin/Streptomycin/Amphotericinβ, PSA) (Biological 03-033-1B), trypsin-EDTA (Biological 03-051-5B), 2′,7′-dichlorofluorescein diacetate (DCFH-DA ) (Sigma-Aldrich D6883, St. Louis, MO, USA), annexin V-FITC fluorescence microscopy kit (BD Pharmingen 550911), propidium iodide staining solution (PI) (BD Pharmingen 556463), and 1,1’-Dioctadecyl-3,3,3’,3’-tetramethylindocarbocyanine perchlorate (DiI) (Sigma 468495).

### 2.2. MB-Liposome Synthesis

DSPC (8 mg) and DPPE-PCB (2 mg) were dissolved in a mixed solvent of 2 mL chloroform and 1 mL methanol, and the solvent was removed under the pressure of 10,000 Pa (100 mbar) overnight to form lipid film. 0.5 mM MB solution (4 mL) in phosphate buffered saline (PBS) at 65 °C was added to dissolve the lipids, then the solution was rotated by rotary evaporation system (Rotavapor, R30, Buchi, Switzerland) at atmospheric pressure in a water bath at 65 °C for an hour, followed by a 21-time extrusion via membrane with a pore size of 200 nm. To eliminate non-encapsulated MB, the solution was dialyzed for 6 hours, with at least 3 dialyzate changes. The concentration of MB in MB-liposome solution was quantified by the absorbance at the wavelength of 660 nm and calibration curve of free MB.

### 2.3. MB Release Test

The prepared MB-liposome solution (2 mL) was loaded in the dialysis membrane (MWCO 12,000–14,000), and dialyzed in 1 L of deionized (DI) water. At each selected time, 0.1 mL of MB-liposome solution was removed and mixed with 2.9 mL of 5% SDS and quantified by the absorbance at the wavelength of 660 nm.

### 2.4. ROS Generation: RNO Test

A volume of 200 μL of 4 μM MB sample (free MB or MB-liposomes solution), 8 μL of 12.5 mM RNO, and 16 μL of 25 mM imidazole were mixed and added into a 96-well plate, and 0/3/6/10/20 min of light exposures (165 mW) at a wavelength of 660 nm were applied. A volume of 150 μL of light exposed solution was diluted with 2.85 mL DI water to determine the absorbance at a wavelength of 440 nm by UV–Vis spectrophotometer.

### 2.5. Cell Culture

Breast cancer cells (4T1 cells) were cultured in DMEM-HG culture medium with 10% FBS and 1% PSA. A total of 1 × 10^6^ cells were first cultured in 10-cm dish at 37 °C and 5% CO_2_. After 24 h cultivation, cells were washed by PBS, and then trypsin-EDTA was applied to detach the cells. The suspended cells were moved to centrifuge tube and centrifuged at 900 rpm for 5 min, and the supernatant fluid were removed. The cells were resuspended with 3 mL culture medium and counted for further experiments.

### 2.6. Cytotoxicity

The 4T1 cells purchased from American Type Culture Collection (ATCC) were seeded at a density of 6500 cells per well in a 96-well culture plate for 24 h. After being washed by PBS once, 100 μL of culture medium with desired concentrations of free MB or MB-liposomes was added to each well. We first diluted the MB-liposome solution to 16 μM MB by sterilized DI water. Then, we mixed two-time concentrated medium and 16 μM MB-liposome at a ratio of 1:1 to get 8 μM MB-liposome solution in the medium. For groups without the light exposure, MTT assay was done after 24 h. For PDT treatment, the cells interacted with each sample for 2 h and were then exposed to 660 nm light (165 mW) for 6 min. After 22 h incubation, MTT assay was applied.

### 2.7. Live and Dead Staining

A total of 6500 cells were cultivated in a 96-well culture plate at a density of 6500 cells per well for 24 h. After cultivated with different concentration of free MB or MB-liposomes for 2 h, PDT groups were exposed to 660 nm light for 6 min and further cultivated for 22 h. A volume of 3.5 μL of PI, 3.5 μL of calcium AM, and 6 mL of PBS were mixed to form a working solution. After washing with PBS, 50 μL of the working solution was added to each well and incubated for 30 min. Finally, the cells were washed once and rinsed with PBS, and the fluorescence images were observed with fluorescence microscope.

### 2.8. Intracellular Uptake

DiI was first dissolved in 99% alcohol at 2.5 mg/mL to be used as a stock solution. Then, 8 mg DSPC and 2 mg DPPE-PCB were dissolved in a mixed solvent of 2 mL chloroform and 1 mL methanol. A volume of 16 μL of DiI stock solution was added and well dispersed. Then the solvent was removed under the pressure of 100 mbar overnight to form lipid film with DiI. A volume of 4 mL of 0.5 mM MB solution at 65 °C was added to dissolve the lipids, then the solution was rotated by rotary evaporation system (Rotavapor, R30, Buchi, Switzerland) at atmospheric pressure in a water bath at 65 °C for an hour, followed by a 21-time extrusion via membrane with a pore size of 200 nm. To eliminate the non-encapsulated MB, the solution was dialyzed for 6 hours, with at least 3 dialyzate changes. After MB quantification with UV–Vis spectrophotometer, 100 μL of DiI-MB-liposomes at the concentration of 8 μM MB were added to a 96-well plate where 4T1 cells were cultivated for 24 h. Fluorescence images were observed at 0/0.5/1/2/4 h post-treatment.

### 2.9. In Vitro ROS Generation: DCFH-DA Assay

A stock solution of 1 mM DCFH-DA was first prepared in DMSO and was diluted to 40 μM with PBS. Then, 4T1 cells were seeded at a density of 6500 cells per well in a 96-well culture plate for 24 h. The PDT groups were treated with different concentrations of free MB or MB-liposomes for 2 h, followed by an exposure to 660 nm light for 6 min. Then the cells were washed with PBS once, and 100 μL of 40 μM DCFH-DA was added to the cells. After 30 min incubation, the cells were washed once with PBS. The images were then observed by fluorescence microscope.

### 2.10. Identification of Cell Death Pathway 

The medium with 8 μM of free MB or MB-liposomes was added and incubated for 2 h, and exposed to 660 nm light for 6 min. After incubated for 22 h, the medium was removed, and each well was washed with PBS twice and then rinsed by annexin V binding buffer, which is diluted from 10× annexin V binding buffer with DI water. Annexin V-FITC, PI, and annexin V binding buffer were mixed at a ratio of 5:1:45 by volume to prepare the working solution. A volume of 100 μL of working solution was added to each well. After 15 min, the cells were washed twice with annexin V binding buffer and observed with fluorescence microscope.

### 2.11. In Vivo Toxicity Test

Free MB or MB-liposomes were injected in male ICR mice (6 weeks old) via tail vein at a concentration of 1 mg MB/kg and a total volume of 100 μL [3,44,45,46]. The body weights were recorded for 14 days after injection.

### 2.12. Statistical Analysis

All data are expressed as means ± standard deviation. Comparison of different groups was determined using ANOVA, and a significant difference was assumed to be a *p*-value ≤ 0.05. In the following content, “n” represents replicate culture, and “N” represents independent experiments.

## 3. Results and Discussion

### 3.1. Characterization of MB-Liposome

**Figure 2:**
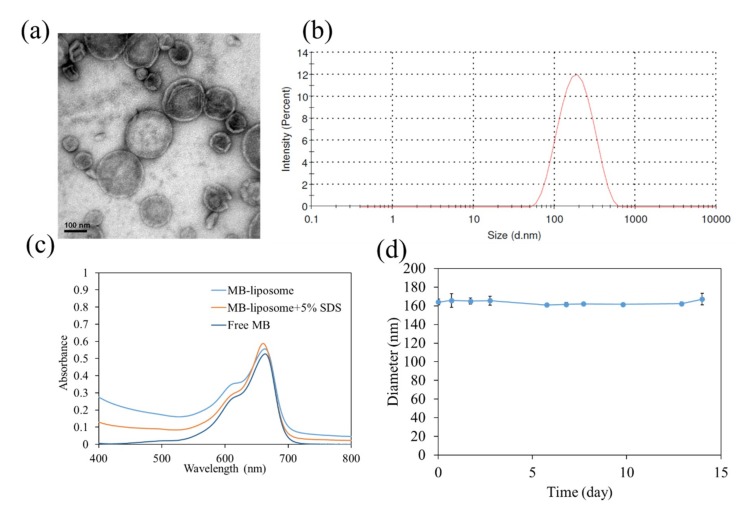
(**a**) The TEM image of Methylene blue (MB)-liposomes.; (**b**) Dynamic light scattering (DLS) measurement of MB-liposomes.; (**c**) UV–Vis spectra of free MB, MB-liposomes, and MB-liposomes with 5% sodium dodecyl sulfate (SDS).; (**d**) hydrodynamic diameter of MB-liposomes over 14 days.

After the synthesis process, we first confirmed the structure and size of liposomes via transmission electron microscope (TEM) and dynamic light scattering (DLS). The shrink and wrinkle represented the film-like structure of liposomes, which were caused by the dehydration process for TEM (Figure 2a and Figure S2). The diameter of liposomes was approximately 140 ± 35 nm (over 50 counts). The hydrodynamic diameter measured by DLS was about 163 nm (Figure 2b). The diameters determined by TEM and DLS are around 150 nm, since liposomes had undergone an extrusion process through a membrane with 200 nm pore size. The particle size determined by DLS was slightly higher than that determined by TEM because of the interaction between water molecules and the slightly charged lipid. The UV–Vis spectra are shown in Figure 2c. The absorption peak at 660 nm represented methylene blue. After 5% SDS was added, the absorbance at 450 to 550 nm decreased, which can be assumed as the breakdown of liposomes. Additionally, the absorbance at 660 nm representing MB monomer increased, and the absorbance at 610 nm representing MB dimer decreased. This observation could be attributed to the breakdown of liposomes, so that MB were released from them.

For the stability measurement, the sizes of liposome were determined by dynamic light scattering (DLS) for 14 days (Figure 2d). The size was around 160 nm, and the polydispersity index (PdI) of measurements were low, which indicated liposomes were highly stable (Table S1). In addition, the MB release profile was determined (Figure S3). The MB release was fast, with approximately 95% of MB being released in 8 h.

### 3.2. ROS Generation Ability

**Figure 3:**
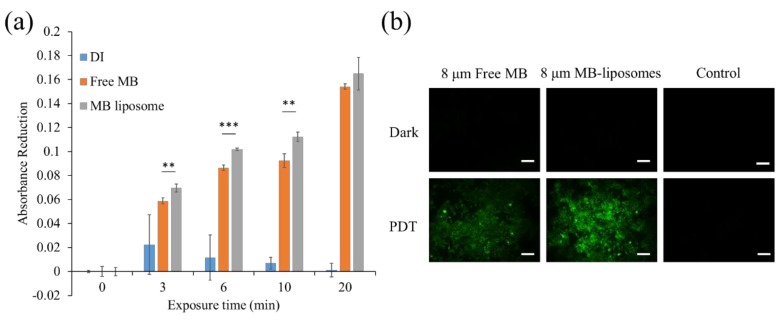
(**a**) *N*,*N*-dimethyl-4-nitrosoaniline (RNO) absorbance reduction at 440 nm of free MB and MB-liposomes after different irradiation time (*n* = 4, **: *p* < 0.01, ***: *p* < 0.001 comparison between free MB and MB liposome at the same exposure time); (**b**) fluorescence images of 2′,7′-dichlorofluorescein diacetate (DCFH-DA) assay of free MB and MB-liposomes. Green, 2′,7′-dichlorofluorescein (DCF); Scale bar, 200 μm.

To determine the ROS generation ability of MB-liposomes after different exposure time (3–30 min), RNO and imidazole were used. Singlet oxygen can first react with imidazole and form imidazole endoperoxide, causing RNO bleaching [47]. The absorbance reduction at the wavelength of 440 nm was shown in Figure 3a. DI water was applied as a control. Results showed that the absorbance reduction of MB-liposomes was slightly higher than free MB, which represented no decrease in the ROS generation ability after the MB got capsulated in the liposomes. The ROS generation of MB was usually maintained after encapsulation or loaded on nanocarriers, and it was also confirmed by another research [19].

Furthermore, to prove that the ROS could also be generated in vitro, DCFH-DA assay was applied. The DCFH-DA is a fluorescence precursor with great permeability through cell membranes. After penetrating the cell membrane, cellular esterase hydrolyzed DCFH-DA into 2′,7′-dichlorodihydrofluorescein (DCFH). The DCFH can further react with ROS and form 2′,7′-dichlorofluorescein (DCF), which fluoresces green [48]. The ROS generation after 6 min of light exposure was monitored via fluorescence microscope. For the three different groups without light exposure, there was no green fluorescence (Figure 3b). After the light exposure, the control group still showed no fluorescence. On the contrary, strong fluorescence was observed in MB-liposomes, which indicated that the cells experienced a high oxidative stress. However, the green fluorescence expressed in free MB was much lesser than that in MB-liposomes.

### 3.3. In Vitro Cytotoxicity Test

**Figure 4:**
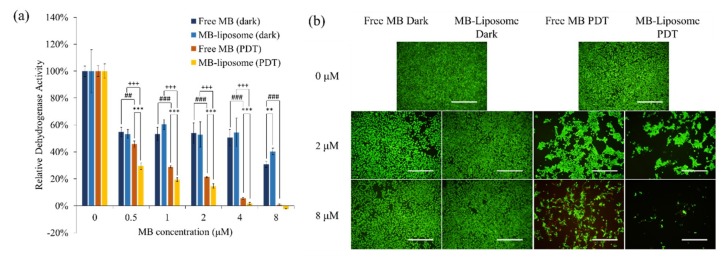
(**a**) The 24-h cell viability (by MTT assay) of free MB and MB-liposomes at different concentrations with dark treatment or photodynamic therapy (PDT) treatment. (*n* = 4, **: *p* < 0.01, ***: *p* < 0.001 comparison between free MB and MB liposome at same concentration of free MB. ##: *p* < 0.01, ###: *p* < 0.001 comparison between dark and PDT treatment at same concentration of free MB. +++: *p* < 0.001 comparison between dark and PDT treatment at same concentration of MB-liposome). (**b**) Live and dead staining after 24 h treatment of free MB and MB-liposomes. Green, calcium acetoxymethyl (AM); Red, PI; Scale bar, 500 μm.

An (3-(4,5-Dimethylthiazol-2-yl)-2,5-diphenyltetrazolium bromide (MTT) assay was performed to analyze the in vitro cytotoxicity of free MB and MB-liposomes, and the results are shown in Figure 4a. The relative dehydrogenase activity of MB-liposomes showed no significant difference from free MB in dark-treated groups; however, at a concentration of 8 μM MB, the MB-liposomes showed a higher relative dehydrogenase activity than the free MB. Although other experiments are required to indicate that liposome capsulation could decrease the toxicity of MB in high concentration, the MTT assay gave a positive result. Moreover, the relative dehydrogenase activity of MB-liposomes treated cells was significantly lower than that of free MB treated cells with light exposure, and also the value decreased significantly with light exposure in both free MB and MB-liposome treated cells. These results were coherent with the in vitro ROS generation examination, which showed a higher ROS generation in MB-liposomes treated cells. The relative dehydrogenase activity also decreased as the MB concentration got higher. Additionally, the live and dead staining demonstrated that the MB-liposomes treated cells showed lower live signal with light exposure, while the cells without light exposure showed more live signal and less dead signal (Figure 4b). In addition, the morphology of cells with light exposure was more spherical, which represented the cells were probably damaged.

### 3.4. Intracellular Uptake

**Figure 5:**
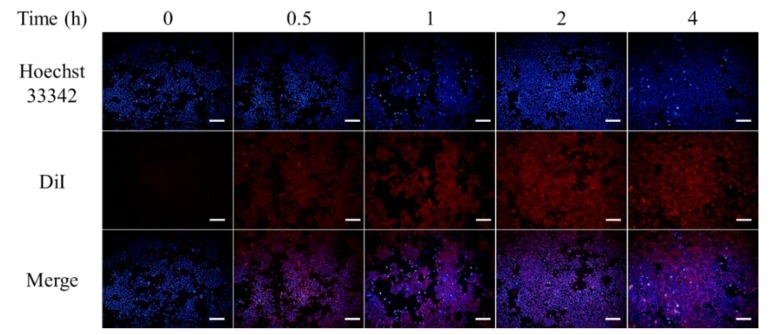
Fluorescence images of cells treated with 1,1′-Dioctadecyl-3,3,3′,3′-tetramethylindocarbocyanine perchlorate (DiI)-MB-liposomes for different time periods. Blue, Hoechst 33342; Red, DiI; Scale bar, 200 μm.

To identify the intracellular uptake profile, cells were cultured with DiI-labelled MB-liposomes for different time periods, and then were stained with Hoechst 33342 to label the nuclei (Figure 5). After 30 min of incubation, the red fluorescence was observed, which indicated the uptake was fast and efficacious, and the relative fluorescence intensity showed that the DiI signal got saturated after 2 h cultivation (Figure S4). Additionally, the blue signal, which represented nuclei, were surrounded by red fluorescence. This observation suggested that DiI, carried by liposomes, got co-localized with the cell membrane, which confirmed the uptake of liposomes.

The result of intracellular uptake offered a reasonable explanation of the different ROS signal expression in RNO test and DCFH-DA assay. From the RNO test, we indicated that free MB had a similar ROS generation ability to MB-liposomes, but the in vitro ROS generation ability of MB-liposomes was much higher, which was determined by the DCFH-DA assay. This could be attributed to the quickened MB uptake by encapsulating MB into liposomes, causing a higher concentration of MB in cells, in turn leading to a higher ROS generation.

### 3.5. Identification of Cell Death Pathway

Since various regulatory pathways include ROS, the levels of ROS in cells are maintained in a certain region under normal condition. On account of the special character of ROS, including activating cell-cycle inhibitor and inducing cell death pathway, a high level of ROS is unbearable for cells [12,13]. To identify the cell death pathway, cells after different treatments were stained with Annexin V-FITC, which could stain the inversion of phosphatidylserine, and PI, which could bind to the DNA [49].

**Figure 6:**
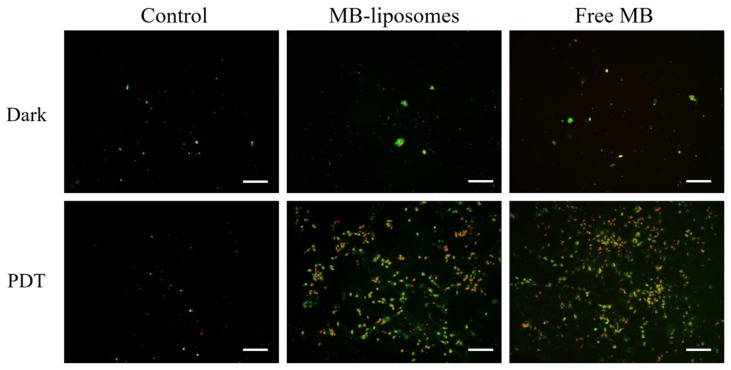
Fluorescence images of Annexin V-FITC (green) and PI (red) after 24 h treatment. Scale bar, 200 μm.

After Annexin V-FITC and PI staining, each group was observed via fluorescence microscopy. The results are shown in Figure 6. The cells with positive Annexin V-FITC but negative PI represented early stage apoptosis, because cell membrane inversion had already been detected. On the other hand, cells with positive PI signal represented late stage apoptosis. Without irradiation, all the images showed sporadic green dots, which indicated that most cells were not damaged, and only a few cells were undergoing early stage apoptosis (Figure 6a–c). The cells after irradiation but no MB treated were still not damaged, since the green and red signals were not obvious (Figure 6d). However, cells cultured with free MB or MB-liposomes expressed both green and red signals after irradiation, which could be attributed to the ROS generated by MB (Figure 6e,f). The observation confirmed that cells with MB and irradiation were triggered to death via apoptosis.

### 3.6. Survival Rate of Mice Administrated with MB-Liposomes

To preliminarily identify the in vivo toxicity, free MB and MB-liposomes were injected in mice via the tail vein at a concentration of 1 mg MB/kg, while PBS-treated mice were taken as the control group (Figure S5). After 14 days, all the mice in the different groups were still alive and ingested normally. The body weight of the free MB and MB-liposome treated mice increased steadily and showed no significant difference to PBS control group. The result could be a sign that the MB-liposome showed no in vivo toxicity, but further experiments should be carried out to confirm the low toxicity.

## 4. Conclusions

In this study, we developed a photodynamic nanoagent as a cancer treatment with high in vitro ROS yield and no in vivo toxicity. First, we successfully encapsulated MB, a well-known phenothiazine, into DPPE-PCB/DSPC liposomes. The size and structure of MB-liposomes were confirmed by DLS and TEM, which was around 160 nm. Additionally, the high stability was also verified by DLS measurements for 14 days. The ROS generation ability was determined by RNO test and DCFH-DA assay, which indicated that MB-liposomes could generate more ROS in vitro than free MB. We also gave this observation a confirmation by performing intracellular uptake test with DiI-labelled MB-liposomes. The cytotoxicity of free MB and MB-liposomes were measured by MTT assay and live and dead staining, which pointed out that MB-liposomes were able to cause more cell death than free MB. Moreover, the cell death pathway was also identified by Annexin V-FITC and PI. Finally, we performed an in vivo experiment by tail vein injection of mice, and the results proved that MB-liposomes had no in vivo toxicity. However, the dark cytotoxicity of MB-liposome solution should be improved. Further optimization of the protocol is needed, such as a lower toxicity drug choice or surface modification. This research can be considered a conceptual research to prove the effect of liposomes as drug carriers.

## Supplementary Materials

The following are available online at http://www.mdpi.com/2079-4991/9/1/14/s1, Figure S1: Structure of DPPE-PCB (poly(carboxybetaine) modified 1,2-dipalmitoyl-sn-glycero-3-phosphoethanolamine); Figure S2: Transmission electron microscope (TEM) image of MB-liposomes in a larger view. MB, methylene blue; Figure S3: MB release profile (*n* = 3); Figure S4: Relative DiI fluorescence intensity to different culture time (*n* = 3). DiI, 1,1’-Dioctadecyl-3,3,3’,3’-tetramethylindocarbocyanine perchlorate; Figure S5: Mice body weights of post-injection over 14 days (*N* = 3); Table S1: Average diameter and PdI of stability profile.
