# Supplementary material for: Methylene-Blue-Encapsulated Liposomes as Photodynamic Therapy Nano Agents for Breast Cancer Cells
Source: Nanomaterials (Basel). 2018 Dec 23;9(1):14. doi: 10.3390/nano9010014 (PMC6359461; doi:10.3390/nano9010014)
Supplement: Supplementary file 1 [file nanomaterials-09-00014-s001.pdf]

nanomaterials

Article

Methylene-Blue-Encapsulated Liposomes as Photodynamic Therapy Nano Agents for Breast Cancer Cells

Po-Ting Wu, Chih-Ling Lin, Che-Wei Lin, Ning-Chu Chang, Wei-Bor Tsai * and Jiashing Yu *

Department of Chemical Engineering, National Taiwan University, Taipei 103, Taiwan; r05524112@ntu.edu.tw (P.-T.W.); r06524045@ntu.edu.tw (C. -L.L.); d01642002@ntu.edu.tw (C. -W.L.); r05524045@ntu.edu.tw (N.-C.C.)

* Correspondence: weibortsai@ntu.edu.tw (W.-B.T.); jiayu@ntu.edu.tw (J.Y.); Tel.: +886-2-3366-9477 (J. Y.)

Received: 17 November 2018; Accepted: 18 December 2018; Published: date

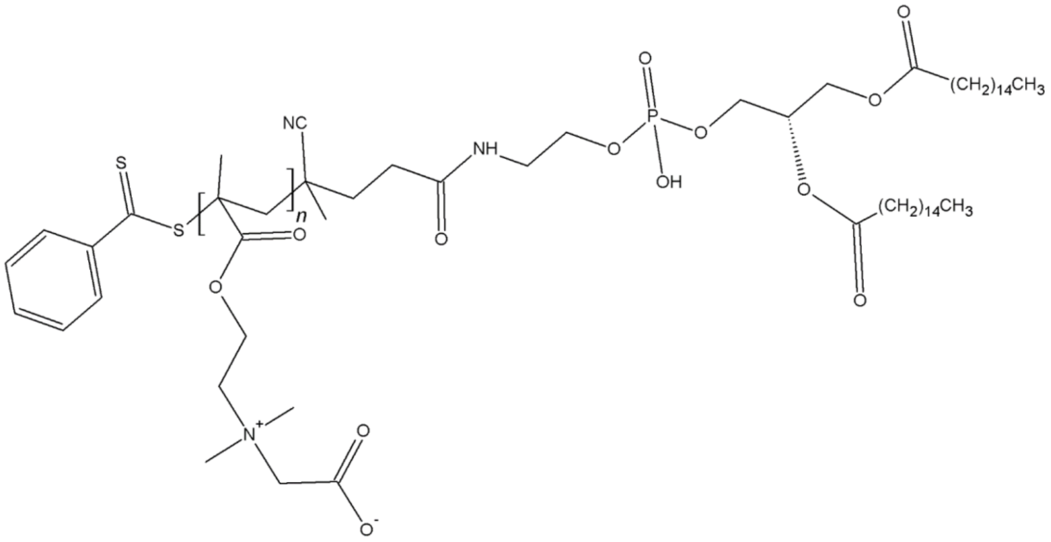

Figure S1. Structure of DPPE-PCB.

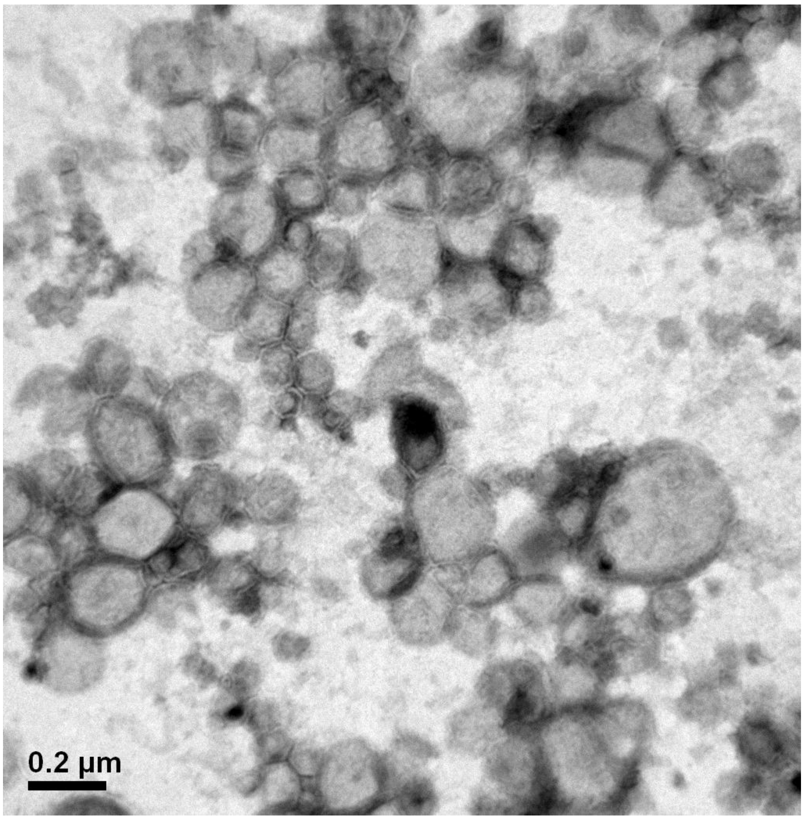

Figure S2. TEM image of MB-liposomes in a larger view.

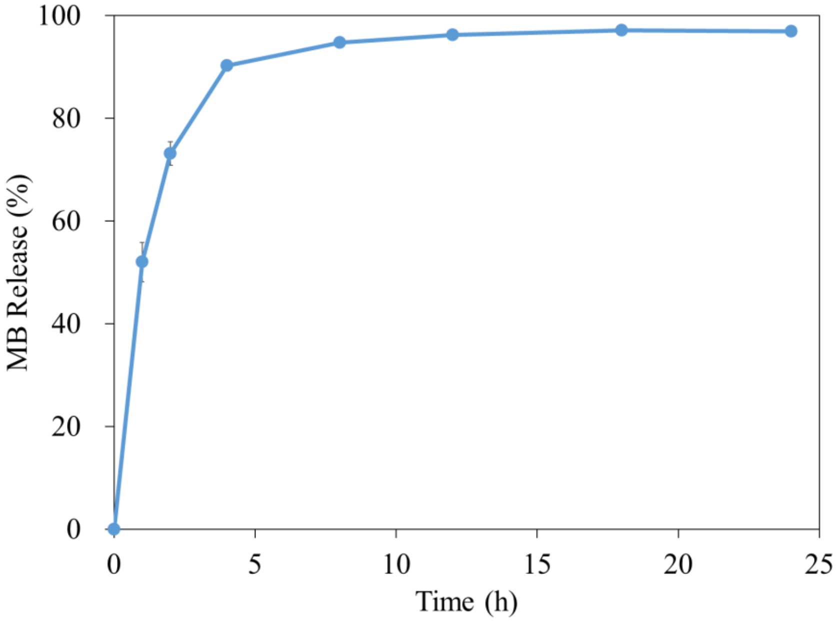

Figure S3. MB release profile. (n=3)

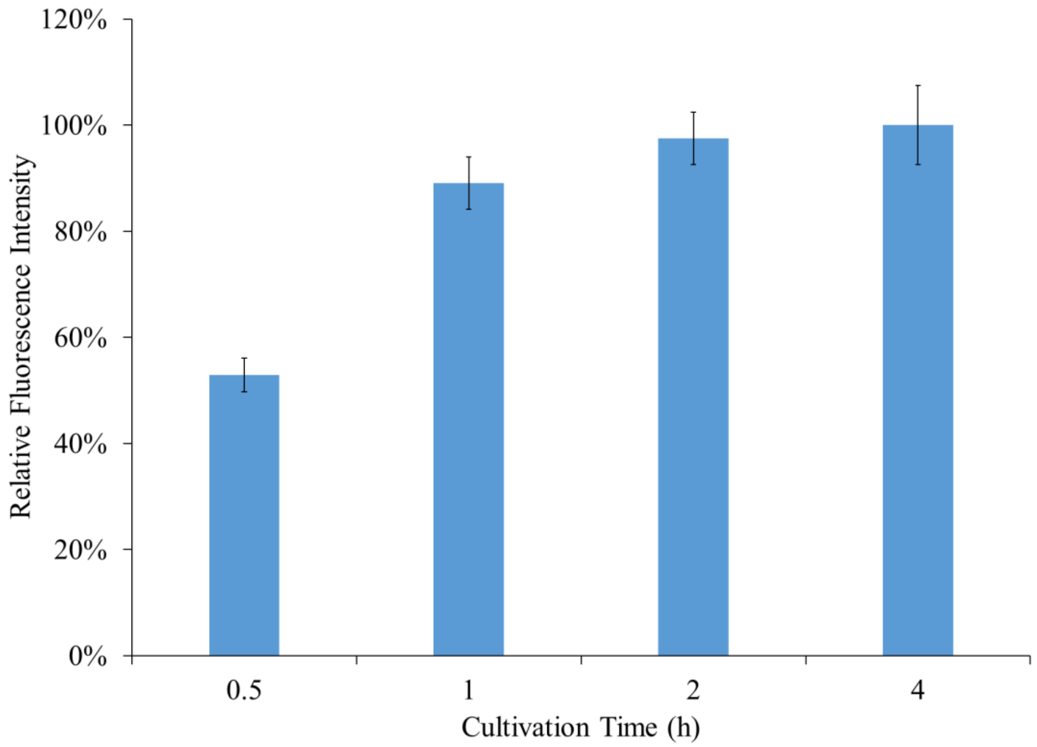

Figure S4. Relative DiI fluorescence intensity to different culture time. (n=3)

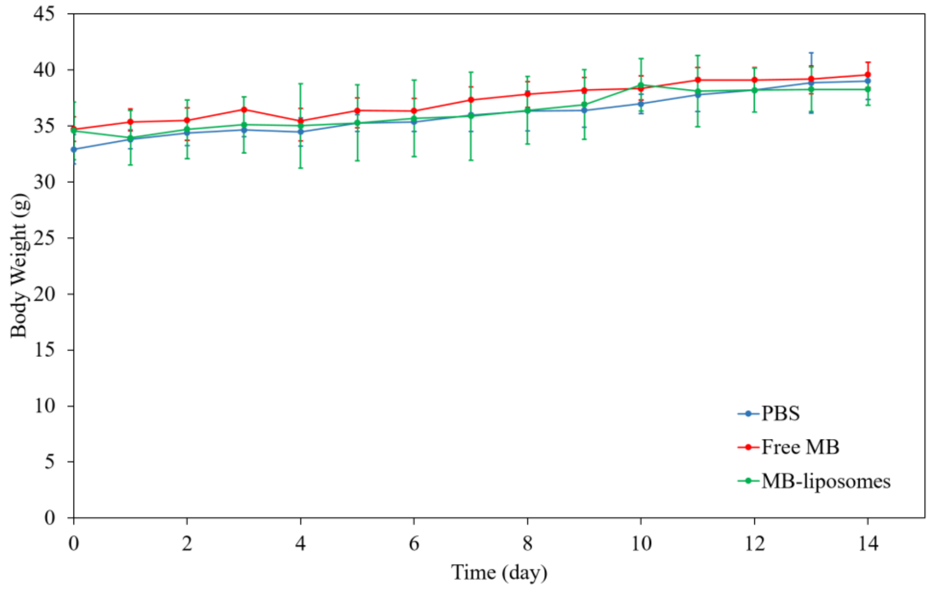

Figure S5. Mice body weights of post-injection in 14 days. (N=3)

Table S1. Average diameter and PdI of stability profile.

Time (h)	Time (day)	Average diameter (nm)	Standard deviation (nm)	PdI
0	0	164.2	3.02	0.174
17	0.708	165.9	7.28	0.170
41	1.708	165.0	3.25	0.167
66	2.750	165.5	4.78	0.141
138	5.750	160.9	1.39	0.114
163	6.792	161.7	2.05	0.136
185	7.708	162.2	1.35	0.138
235	9.792	161.6	1.07	0.111
310	12.917	162.4	1.63	0.131
336	14.000	167.3	6.32	0.151
